# Operative Zugangswege und Implantatwahl im Bereich des Klavikulaschafts

**DOI:** 10.1007/s00113-024-01470-w

**Published:** 2024-08-27

**Authors:** Yannic Lecoultre, Bryan J. M. van de Wall, Frank J. P. Beeres, Reto Babst

**Affiliations:** 1 Klinik für Orthopädie und Unfallchirurgie, Luzerner Kantonsspital, Spitalstrasse, 6000 Luzern, Schweiz; 2 Fakultät für Gesundheitswissenschaften und Medizin, Universität Luzern, Luzern, Schweiz

**Keywords:** Klaviculaschaft, Operative Therapie, Implantatwahl, Plattenosteosynthese, Marknagelosteosynthese, Clavicular shaft, Operative treatment, Implant selection, Plate fixation, Endomedullary nail fixation

## Abstract

**Hintergrund:**

Klavikulafrakturen gehören zu den häufigsten Verletzungen des Schultergürtels. Nichtdislozierte Frakturen werden i. Allg. konservativ behandelt, während dislozierte Frakturen eine chirurgische Versorgung erfordern. Hierfür stehen verschiedene Implantate und Operationstechniken mit zuverlässigen Ergebnissen zur Verfügung. Hauptnachteil sind die häufigen Materialirritationen mit entsprechend hohen Zweiteingriffsraten zur Materialentfernung.

**Ziel der Arbeit:**

Es werden die verschiedenen Operationstechniken für Klavikulaschaftfrakturen mit ihren spezifischen Anwendungsgebieten sowie Vor- und Nachteilen vorgestellt. Diese Übersicht bietet eine Entscheidungshilfe, welche Operationstechnik aufgrund der jeweiligen morphologischen Frakturmerkmale am besten geeignet ist. Darüber hinaus wird ein Überblick über die aktuellen Forschungsaktivitäten gegeben. Ein besonderer Schwerpunkt liegt auf neuen Implantaten, die dazu beitragen könnten, Implantatirritationen zu verringern.

**Ergebnisse und Schlussfolgerungen:**

Die offene superiore und die anteroinferiore Plattenosteosynthese zeigen jeweils ähnliche zuverlässige Ergebnisse. Die Technik der minimalinvasiven Plattenosteosynthese (MIPO) bietet eine Alternative für multifragmentäre Frakturen; hier weist sie im Vergleich zum offenen Verfahren eine geringere Komplikationsrate auf. Die Doppelplattenosteosynthese mit Minifragmentplatten erzielt vielversprechende Ergebnisse in Bezug auf die implantatbedingten Irritationen. Größere prospektive Studien stehen noch aus. Die Marknagelung ist eine gute Alternative, v. a., wenn ohnehin eine Materialentfernung geplant ist, z. B. im pädiatrischen Setting.

## Abstract (en)

**Background:**

Clavicle fractures are among the most frequent injuries of the shoulder girdle. Nondisplaced fractures are generally treated conservatively, whereas dislocated fractures require surgical reduction and stabilization. A variety of implants and surgical techniques with reliable results are available. While all techniques provide similar healing rates, they share a common disadvantage with the high incidence of implant irritation and correspondingly high rates of second interventions for material removal.

**Objective:**

The various surgical techniques for clavicle shaft fractures with their specific areas of application as well as advantages and disadvantages are presented. This review also provides an aid for deciding which surgical technique is most appropriate based on the fracture morphology. Furthermore, an overview of current research activities is presented, with a specific focus on new implants which could help to reduce implant irritation.

**Results and conclusion:**

Open superior and anteroinferior plate osteosyntheses each show similar reliable results. The minimally invasive plate osteosynthesis (MIPO) technique offers an alternative for multifragmented fractures as it has a lower complication rate compared to the open procedure. Double plate osteosynthesis using minifragment plates shows promising results with respect to the incidence of implant-related irritation. Larger prospective studies are still pending. Intramedullary nailing offers a good alternative, especially if material removal is planned anyway, e.g., in the pediatric setting.

Klavikulafrakturen gehören zu den häufigsten Verletzungen des Schultergürtels. Die konservative Therapie war lange Standard. Aufgrund ihrer verminderten Komplikationsraten und verkürzten Rekonvaleszenzzeit wuchs der Stellenwert der operativen Stabilisierung zunehmend. Zur operativen Versorgung stehen unterschiedliche Verfahren mit zuverlässigen Ergebnissen zur Verfügung, diese sind jedoch mit hohen Zweiteingriffsraten, insbesondere aufgrund der Materialentfernung, verbunden. Ziel der aktuellen Forschung ist die Verringerung der Reoperationen ohne Kompromittierung der Heilungs- und Komplikationsraten.

## Hintergrund

Klavikulafrakturen gehören zu den häufigsten Frakturen des menschlichen Körpers. Aufgrund der geringen muskuloligamentären Stabilisierung im Schaftbereich und der geringen Weichteilüberdeckung ist die Klavikula besonders anfällig für Traumata. Diese Frakturen machen rund 5 % aller Frakturen beim Erwachsenen aus, wovon rund 70 % auf den Schaftbereich entfallen. Sie treten gehäuft bei jungen, sportlich aktiven Patienten mit hohem funktionellem Anspruch auf, und es zeigt sich eine zyklische zeitliche Verteilung mit Maximum im Frühjahr und im Herbst während der Radsportsaison [25].

Eine konservative Therapie ist grundsätzlich auch bei dislozierten Frakturen möglich und war über Jahrzehnte der Standard. Diese führte aber zum vermehrten Auftreten von Non- bzw. Malunion in 14,5 bzw. 8,5 % der Fälle und zum verzögerten Wiedererlangen der Funktion [4]. Die Ergebnisse nach konservativer Therapie in Kombination mit der Patientendemografie und deren Bedürfnissen begründet die Verlagerung der Therapie in Richtung einer operativen Stabilisierung [15].

Nach konservativer Therapie sind Nonunion-Raten erhöht und das Wiedererlangen der Funktion verzögert

Es existieren verschiedene Verfahren, die sich in Bezug auf Heilung, Funktion und Komplikationen weitgehend als gleichwertig erwiesen haben [6]. Aufgrund der geringen Weichteilüberdeckung ist allen Verfahren eine hohe Rate an Zweiteingriffen aufgrund von störendem Osteosynthesematerial gemeinsam. Dies verursacht hohe Kosten (im Setting der Autoren mindestens 1500 CHF allein für die Operation, die volkswirtschaftlichen Kosten nicht eingerechnet) und ist nicht ohne Komplikationsrisiko. Vor allem ist eine erhöhte Refrakturrate von ca. 5 % zu erwähnen [26]. Die Therapiewahl ist sowohl vom Frakturbild, von den Patientenbedürfnissen und den Frakturlokalisation als auch von der Erfahrung und den Präferenzen des Behandlungsteams abhängig.

## Klassifikation

Die gebräuchlichste Einteilung der Klavikulafraktur ist die Klassifikation nach Allmann, für die die Lokalisation der Fraktur (laterales, mittleres und mediales Drittel) entscheidend ist. Frakturen des mittleren Drittels sind mit mindestens 70 % aufgrund der fehlenden ligamentären Stabilisierung und des geringeren Durchmessers weitaus am häufigsten. Diese Schaftfrakturen können gemäß ihrem Frakturmuster weiter in einfache, Biegungskeil- oder multifragmentäre Frakturen unterteilt und mithilfe der AO-Klassifikation gut reproduzierbar beschrieben werden (Typ A: einfach, Typ B: Biegungskeil, Typ C: multifragmentär, [1, 16]).

Durch den Zug des Sternokleidomastoideus entsteht typischerweise eine Dislokation des medialen Fragments nach kranial, während das laterale Fragment von den korakoklavikulären Bändern in Position gehalten, durch das Gewicht des Arms nach kaudal und durch die Pektoralmuskulatur nach medial gezogen wird.

## Indikation zur Operation

Während in der Vergangenheit der Großteil der Frakturen konservativ behandelt wurde, hat sich dieses Gleichgewicht in den letzten 10 bis 15 Jahren deutlich zugunsten der Operation verschoben [4, 15].

Undislozierte Frakturen werden grundsätzlich weiterhin konservativ behandelt. Eine Operationsindikation besteht bei aktiven Patienten mit um mehr als Schaftbreite verschobenen oder über 2 cm verkürzten Frakturen sowie bei neurovaskulärer Kompromittierung oder Hautgefährdung. Dazwischen ist ein Graubereich, in dem beide Therapieoptionen Anwendung finden. Für die Entscheidungsfindung sind Patientenfaktoren wie Alter, Komorbiditäten und der funktionelle Anspruch hilfreich. Bei hohem Anspruch und akzeptablem perioperativem Risiko wird heute tendenziell aufgrund einer geringeren Nonunion-Rate, früherer Konsolidierung und früherem Wiedererlangen der vollen Funktion der operativen Versorgung der Vorzug gegeben [6].

## Zugangswege und Implantate

Der operative Zugang zur Klavikula ist verhältnismäßig unkompliziert und richtet sich nach der Lokalisation und den morphologischen Merkmalen der Fraktur bzw. nach dem gewählten Implantat. Es stehen diverse offene und minimalinvasive Techniken für eine Plattenosteosynthese und für die Fixierung mithilfe des Marknagels zur Verfügung; diese haben jeweils individuelle Indikationen mit ihren Vor- und Nachteilen.

### Superiore Plattenosteosynthese

Die offene Reposition und Osteosynthese mithilfe der superioren Plattenlage eignet sich für Frakturen der AO-Typen A–C; insbesondere bei einfachen Frakturen sollen die anatomische Reposition und absolute Stabilität eine direkte Frakturheilung ermöglichen.

Die Lagerung erfolgt in Beach-Chair- oder Rückenlage; der Arm des Patienten wird zur einfacheren Frakturreposition frei abgedeckt. Der Hautschnitt erfolgt entweder direkt anterosuperior entlang der Klavikula, schräg entlang der Hautspaltlinien oder als „coup de sabre“ senkrecht. Die schräge Inzision innerhalb der Hautspaltlinien hat sich im Hinblick auf Patientenzufriedenheit, Kosmetik, Blutverlust und Schädigung der supraklavikulären Nervenäste als überlegen gezeigt. Letztere ist jedoch häufig zumindest teilweise reversibel und scheint kaum einen Einfluss auf die Patientenzufriedenheit zu haben [14, 28].

Anschließend wird unter vorsichtiger Blutstillung durch das Platysma direkt auf den Knochen, möglichst unter Schonung der supraklavikulären Nervenäste, präpariert, die Fraktur dargestellt und reponiert [2].

Zur Stabilisierung steht eine Vielzahl von Implantaten zur Verfügung, darunter winkelstabile 3,5-mm-Kompressionsplatten, anatomisch präformierte winkelstabile Implantate mit 2,7–3,5 mm sowie 3,5-mm-Rekonstruktionsplatten, die allerdings eine höhere Versagensrate aufweisen [6, 27]. Diese werden mit mindestens 3 Schrauben/Frakturseite besetzt. In den letzten Jahren werden vermehrt weniger auftragende („Low-profile“-)Formplatten verwendet [6, 25].

Biomechanisch hat sich eine erhöhte Steifigkeit durch die Nutzung von winkelstabilen Implantaten gezeigt, insbesondere wenn anatomisch präformierte Platten genutzt werden. Uchiyama et al. konnten in einer Studie mit 102 Patienten eine deutlich reduzierte Konsolidierungszeit (13 vs. 17,5 Wochen) zeigen [20, 23].

Die Erfolgsraten bei superiorer Plattenlage sind unter Beachtung der AO-Prinzipien zuverlässig hoch: Die Heilungsraten betragen 98–99 %, und es wird meist eine uneingeschränkte Funktion mit über 90 %iger Rückkehr in die frühere sportliche Tätigkeit beschrieben [6]. Die Häufigkeiten von Komplikationen, z. B. durch Materialversagen, Infektionen oder Wundheilungsstörungen, bewegen sich je nach Studie zwischen 0 und 10 % [26].

Die superiore Plattenlage führt häufig zu Irritationen

Die superiore Plattenlage führt, bedingt durch den geringen Weichteilmantel über der Klavikula, häufig zu Irritationen. Dies stört insbesondere aktive Patienten bei sportlichen Tätigkeiten oder beim Tragen von Rucksäcken. Folge sind Materialentfernungsraten zwischen 17 und 70 %, die zudem mit einer bis zu 5 %igen Refrakturrate assoziiert sind [6, 26]. Die notwendigen Zweiteingriffe belasten die finanziellen und personellen Ressourcen des Gesundheitssystems.

### Anteroinferiore Plattenosteosynthese

Als Alternative zur superioren hat sich die anteroinferiore Plattenlage etabliert. Hintergrund sind biomechanische und anatomische Überlegungen: Einerseits sollen aufgrund der abgeflachten Form der Klavikula die Schraubenlänge und der Halt verbessert werden. Außerdem könnte durch die veränderte Bohrrichtung die Gefahr von Gefäß- und Lungenverletzungen verringert werden. Zudem erlaubt diese Plattenlage eine bessere Weichteildeckung [3].

Die Operationstechnik gleicht in weiten Teilen der superioren Plattenosteosynthese, wobei zur Platzierung Teile der pektoralen Muskulatur abgelöst werden müssen. Hierfür stehen ebenfalls anatomisch vorgeformte Implantate sowie Kompressions‑/Verriegelungsplatten- und Rekonstruktionsplatten zur Verfügung.

Biomechanisch unterscheidet sich die anterioinferiorere von der superioren Platte durch eine höhere Kompressions- und Torsionssteifigkeit (799 vs. 616 N/mm bzw. 313 vs. 307 Nmm/Grad) bei tieferer horizontaler Biegungsresistenz (40 vs. 251 N Bruchlast) am Beispiel von 3,5-mm-LCDRC(„Locking Conturable Dual Reconstruction Plate“)-Platten (Zimmer, Inc., Warsaw, IN, USA) [20].

Buenter et al. publizierten kürzlich eine „Natural-experiment“-Studie mit hoher Fallzahl, in der keine Unterschiede der Materialentfernungsraten wegen Weichteilirritationen, Materialversagen oder im Hinblick auf das funktionelle Ergebnis der anterioinferioren gegenüber der superioren Plattenlage gezeigt werden konnten [3, 24].

### Low-profile-Doppelplattenosteosynthese

Ein jüngerer Ansatz mit dem Ziel einer Reduktion von Zweiteingriffen durch Materialentfernungen ist die Stabilisierung der Klavikula mithilfe kleiner dimensionierten und weniger auftragenden Doppelplatten. Anstelle einer voluminöseren, einzelnen Platte werden 2 deutlich kleinere Implantate genutzt. Wegen des geringeren Durchmessers, der besseren Anpassung und der orthogonalen Plattenfixierung wird eine Reduktion der Materialirritation bei ähnlicher biomechanischer Stabilität postuliert. Außerdem soll durch den entsprechend kleineren Zugang eine Schädigung der supraklavikulären Nervenäste reduziert werden [22].

Es wird eine 2,0-mm-Platte superior in Kombination mit einer 2,4- oder 2,7-mm-Platte anteroinferior verwendet und mit jeweils 2 bis 3 Schrauben/Frakturseite besetzt. Der Zugang gleicht dem oben beschriebenen für die Einzelplattenosteosynthese; der Hautschnitt kann aber aufgrund der kürzeren Implantate deutlich geringer ausfallen. Biomechanisch zeigt sich im Kadaverknochen, verglichen mit der Einzelplattenosteosynthese, für dieses Konstrukt eine etwas höhere Steifigkeit bei gleichbleibender Zyklenzahl bis zum Materialversagen [19].

Die Doppelplattenosteosynthese erfolgt mit 2 kleinen Implantaten statt einer voluminösen Einzelplatte

Eine Metaanalyse von Rompen et al. aus dem Jahr 2021 zeigte günstige Ergebnisse hinsichtlich der Reinterventionsrate (6,1 % vs. 16,1 %, *p* = 0,02) im Vergleich zur Einzelplatte, wofür primär die implantatassoziierten Beschwerden verantwortlich waren. Einschränkungen der Heilungsrate oder sonstige Komplikationen, die aufgrund der potenziell größeren Weichteilschädigung postuliert werden, haben sich in dieser Metaanalyse nicht bestätigt [21].

**Abb. 1:**
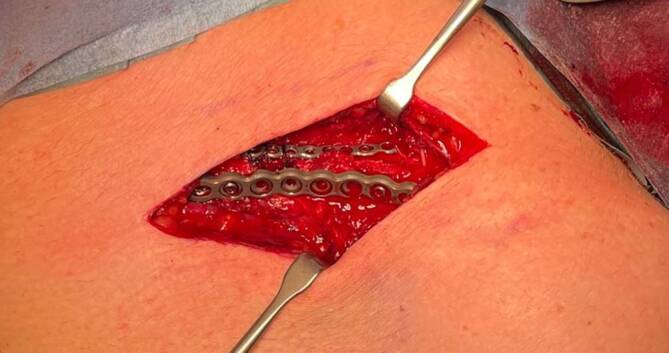
Intraoperative Bilder einer Doppelplattenosteosynthese mit superiorer 2,0-mm- und anteroinferiorer 2,7-mm-Minifragmentplatte

Ähnliche Daten ergaben sich in einer aktuellen, retrospektiven Studie im eigenen Patientenkollektiv mit 99 konsekutiv zwischen 2020 und 2022 behandelten Patienten. Dort zeigen sich neben einer signifikant geringeren Materialentfernungsrate (25,0 vs. 51,7 %) und kürzeren Operationszeit für die Doppelplatte (95 vs. 111 min) keine Unterschiede zwischen den Techniken bezüglich Komplikationen wie Materialversagen oder Nonunion [12]. Eine kürzlich publizierte prospektive randomisierte Studie konnte ebenfalls eine deutliche Reduktion implantatassoziierter Beschwerden nachweisen (27 % vs. 60 %, *p* = 0,03, [11]). In einer derzeit stattfindenden multizentrischen, prospektiven Natural-experiment-Studie wird untersucht, ob die Doppelplattenosteosynthese (Abb. 1) im Vergleich zur superioren oder zur anteroinferioren 2,7-mm-Platte ebenfalls zu einer geringeren Reinterventionsrate beitragen kann. Erste Ergebnisse sind 2025 zu erwarten [13].

Das Ziel ist, die größtmögliche Reduktion von implantatassoziierten Irritationen zu erreichen, ohne die Stabilität und Heilung zu beeinträchtigen.

### Minimalinvasive Plattenosteosynthese

**Abb. 2:**
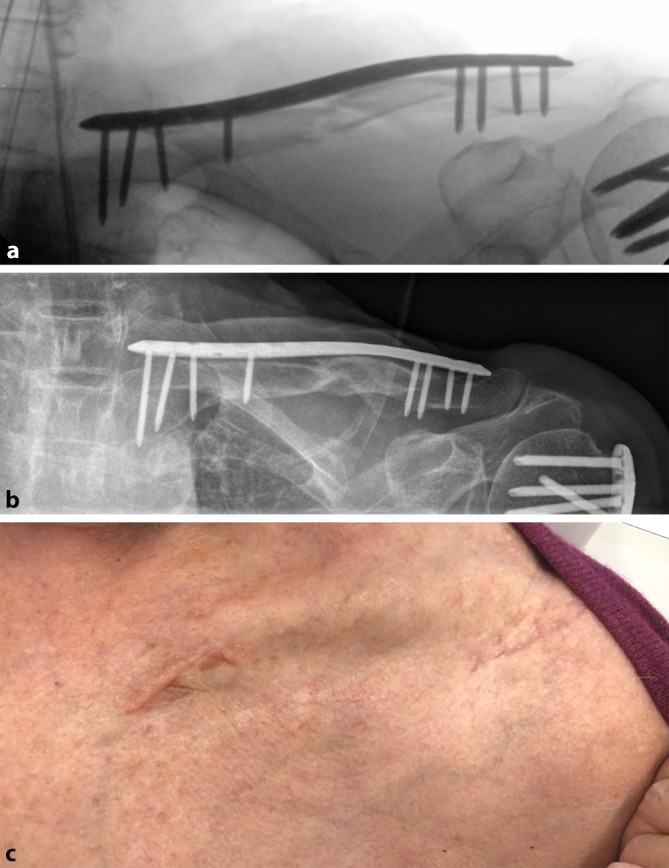
Intraoperatives Kontrollbild (**a**), Röntgenbild ein Jahr postoperativ (**b**) und Operationsnarben 6 Wochen postoperativ (**c**) nach der Versorgung mithilfe einer minimalinvasiven Plattenosteosynthese (MIPO)

Die minimalinvasive Plattenosteosynthese (MIPO; Abb. 2) ist v. a. für multifragmentäre Klavikulafrakturen, u. a. bei kompromittierten Weichteilen, eine gute Alternative. Biomechanisch stabilisiert eine lange, normal dimensionierte Platte, superior oder anteroinferior durch einen lateralen und medialen Zugang eingebracht, als Fixateur interne mehrfragmentäre Frakturmuster in einer Brückenplattenkonfiguration. Der Anspruch an diese Osteosynthese ist nicht die anatomische, sondern die funktionelle Reposition. Die Platte wird durch kurze Inzisionen über dem medialen oder lateralen Ende der Platte epiperiostal eingebracht und durch eine konventionelle Schraube präliminär besetzt. Anschließend erfolgen die Einstellung der Achse und der Länge sowie die Fixierung mit einer zweiten Kortexschraube und danach winkelstabilen Schrauben via Stichinzisionen auf beiden Seiten [17].

Eine Metaanalyse von Zhao et al. ergab keine Unterschiede in Heilungsraten, Funktion und Operationszeit, allerdings eine deutliche Reduktion der Sensibilitätsstörungen über der Klavikula und der Gesamtkomplikationen [29].

### Intramedulläre Nagelung

Als weitere Option der Klavikulaosteosynthese hat sich in den letzten 2 Jahrzehnten v. a. im deutschen Sprachraum die Marknagelosteosynthese mithilfe der elastischen Titanmarknagelung etabliert [9]. Diese wird v. a. für einfache Frakturmuster des mittleren Drittels (AO-Typen A und B) empfohlen. Im Gegensatz zur Plattenosteosynthese hat sie den Vorteil des minimalinvasiven Zugangs und kleinerer, unauffälligerer Narben, eines geringeren Weichteilschadens und niedrigerer Raten von Sensibilitätsstörungen aufgrund der Schonung der supraklavikulären Nervenäste. Typischerweise werden elastische Titanmarknägel in der durch den Markkanal vorgegebenen Dicke (normalerweise zwischen 2,5 und 3,5 mm) verwendet. Aufgrund der vermehrten Materialkomplikationen (z. B. Dislokation der Endkappen, Verbiegung und Materialbrüche) sollte auf Endkappen verzichtet werden [5, 18].

Der Zugang kann via Stichinzision von medial-anteroinferior oder seltener von lateral-posterior erfolgen, wobei der Eintrittspunkt aufgrund der besseren Verankerung im kürzeren Fragment gewählt werden sollte [10]. Der Nagel wird in den Markkanal eingebracht und bis zur Fraktur vorgeschoben. Anschließend wird diese geschlossen oder, wenn nötig, offen reponiert und der Nagel ins gegenüberliegende Fragment eingebracht. Das Nagelende wird mit einem Spezialinstrument so gekürzt, dass das Material entfernt werden kann [10].

In Bezug auf Funktion, Heilung und Komplikationen zeigt die Titanium-Elastic-Nail(TEN)-Osteosynthese bei einfachen Frakturmustern ähnliche Resultate wie die Plattenosteosynthese, allerdings bestehen die Gefahr der Materialmigration sowie eine hohe Rate an Irritationen bis hin zur Hautgefährdung oder Perforation durch das herausragende Nagelende. Eine Materialentfernung ist deshalb in bis zu 80 % der Fälle nach erfolgter Heilung beschrieben. Die TEN-Osteosynthese ist v. a. bei pädiatrischen oder adoleszenten Patienten, bei denen eine Materialentfernung häufig geplant erfolgt, weit verbreitet [7, 8].

## Diskussion

Die Therapie der Klavikulaschaftfraktur hat sich, gestützt auf die Evidenz der letzten Jahrzehnte, in Richtung der operativen Versorgung verlagert. Diese wird durch neue Implantatgenerationen mit sehr zuverlässigen Ergebnissen weiter gefördert. Insbesondere bei jungen, aktiven Patienten wird die Indikation zur operativen Stabilisierung aufgrund der kürzeren Rehabilitationszeit bei tieferen Komplikationsraten großzügig gestellt [4, 15, 25].

Für die Stabilisierung von Klavikulafrakturen stehen unterschiedliche zuverlässige Verfahren mit spezifischen Vor- und Nachteilen zur Verfügung. Die klassische Versorgungstechnik mithilfe superiorer oder anteroinferiorer Formplatte und auch die Marknagelosteosynthese erzielen bei korrekter Anwendungstechnik zuverlässige Resultate mit niedrigen Komplikations- und Heilungsstörungsraten bei einfachen Frakturen, dies jedoch bei einer hohen Reoperationsrate aufgrund von Weichteilirritationen durch das Material. Die MIPO-Technik bietet sich bei multifragmentären Frakturen an. Trotz geringer beschriebener Fallzahlen scheint diese Versorgungstechnik niedrige Komplikationsraten aufzuweisen und sollte bei komplexen Frakturmustern oder Weichteilkompromittierung in Betracht gezogen werden. Für einfachere Frakturmuster könnte die Doppelplattenosteosynthese wegen möglicherweise geringerer Zahl von Sekundäreingriffen durch störendes Implantatmaterial eine vielversprechende Alternative sein. Die diesbezügliche Datenlage ist aber noch gering, um eindeutige Empfehlungen abzugeben.

## Fazit für die Praxis

- Dislozierte Klavikulaschaftfrakturen werden aufgrund der geringeren Komplikationsraten und rascheren Heilung operativ versorgt. Eine konservative Therapie empfiehlt sich nur noch bei geringem funktionellem Anspruch und erhöhtem perioperativem Risiko.
- Hauptnachteil der gängigen Osteosyntheseverfahren sind die hohen Raten der Materialirritationen und konsekutiv Zweiteingriffe zur Materialentfernung.
- Die offene superiore und anterioinferiore Plattenosteosynthese erzielen zuverlässige Ergebnisse bei einfachen und komplexen Frakturen.
- Die MIPO-Technik bietet eine Alternative bei multifragmentären Frakturen; hier weist sie im Vergleich zum offenen Verfahren eine geringere Komplikationsrate auf.
- Bei der Doppelplattenosteosynthese mithilfe von Minifragmentplatten ergeben sich Hinweise auf eine deutlich reduzierte Materialirritation und geringere Metallentfernungsrate. Größere prospektive Studien sind aktuell ausstehend.
- Die Marknagelosteosynthese bietet eine valable Alternative, wenn eine Materialentfernung geplant wird.
